# Gait Symmetry in Children with Autism

**DOI:** 10.1155/2012/576478

**Published:** 2012-05-20

**Authors:** Victoria L. Chester, Matthew Calhoun

**Affiliations:** Andrew and Marjorie McCain Human Performance Laboratory, Richard J. Currie Center, Faculty of Kinesiology, University of New Brunswick, Fredericton, NB, Canada E3B 5A3

## Abstract

Most studies examining gait asymmetry have focused on infants and toddlers and have tended to use subjective methods of evaluating movement. No previous studies have examined gait symmetry in older children with autism using objective motion capture systems. The purpose of this paper was to quantify gait symmetry in children with autism versus age-matched controls. Fourteen children with autism (*N* = 14) and twenty-two (*N* = 22) age, height, and weight-matched controls participated in the study. An eight camera Vicon motion capture system and four Kistler force plates were used to compute temporal-spatial parameters and symmetry indices during walking. Group differences in these measures were tested using MANOVAs. No significant differences between the autism and control group were found for any of the temporal-spatial measures or symmetry indices. Therefore, results suggest that children with autism demonstrate typical symmetry or interlimb movement during gait. Further research is needed to examine the use of different gait inputs to the symmetry indices (e.g., joint angles and moments). A greater awareness of the movement patterns associated with autism may increase our understanding of this disorder and have important implications for treatment planning.

## 1. Introduction

Autism is a developmental disorder that is typically characterized by impaired social interactions and communication and restricted, repetitive, and stereotyped patterns of behaviour [1]. Numerous studies have also found evidence of movement impairments in children with autism. Asymmetry of movements during lying [2–4], righting [3], sitting [2, 5], crawling [2, 3], and standing [6] has been reported during early development. Several studies have also found gait disturbances in children and adults with autism [2, 3, 7–10].

Studies of movement in autism have examined symmetry of movement as an indicator of underlying neurological impairments. Research on movement asymmetries has typically focused on infants and toddlers [3, 11]. Studies of older children and adults with autism have typically described movement impairments but not asymmetry [8, 10, 12–15]. Therefore, it is not known whether movement asymmetries persist with development. Further, most of the above studies on gait symmetry in autism have used observational scales and video analyses. The examination of gait symmetry using motion capture systems may facilitate a more objective analysis of symmetry during gait. The measurement of temporal-spatial data may allow the monitoring of compensations occurring during gait, reflecting an underlying neuromuscular impairment.

There are several symmetry indices that can be used to assess interlimb differences during gait [16–18]. However, there is currently no standardized method of computing gait symmetry. Furthermore, the gait parameters used to compute the various symmetry indices vary across studies. Therefore, the purpose of this study was to examine differences in gait symmetry between a group of children with autism versus age-matched controls using six different symmetry indices and various temporal-spatial measures.

## 2. Method

### 2.1. Participants

The data presented in the study were collected as part of a larger kinematic and kinetic gait study of children with autism [7]. Fourteen children (*n* = 14) aged 5 to 9 years with autism were recruited to participate in the study (mean age = 5.9 yrs; mean height = 120.06 cm; mean weight = 28.67 kg). All of the children had been previously diagnosed with autism spectrum disorder; however, none of the children were diagnosed with Asperger's or otherwise nonspecified disorder. Further participant characteristics are provided in Table 1. Previously published control data [7] was used for data comparisons and consisted of twenty-two (*n* = 22) children aged 5–9 years (mean age = 6.22 yrs; mean height = 119.42 cm; mean weight = 28.66 kg). The control group was matched to the autism group based on age, weight, and height. Children were not matched based on IQ as it is not necessarily an appropriate criterion for matching on experiments involving motor tasks or children with developmental delay. Ethical approval for this study was obtained from the University of New Brunswick Research Ethics Board. Parental consent and child assent were obtained prior to each child's participation in the study.

### 2.2. Instrumentation

An eight camera Vicon MCam motion capture system (Oxford Metrics Group Ltd., Oxford, UK) tracked the three-dimensional trajectories of reflective markers (25 mm diameter) placed on the participants' heel during barefoot walking. Marker trajectories were sampled at a frequency of 60 Hz. Four force plates (Kistler Instruments, Winterthur, Switzerland) were used to measure the three-dimensional forces and moments during gait at a sampling frequency of 600 Hz. The force plates consist of four triaxial transducers at each corner that measure load. The plates were embedded in the laboratory floor and disguised with flooring to prevent targeting by the subjects. Force plates provide information on the interaction of the foot with the floor. As the foot contacts the plate, reaction forces are generated in an equal and opposite direction to the applied load. The examination of these ground reaction forces facilitates the identification of foot contact and lack of contact with the ground. The timing of these force events can then be used to estimate temporal-spatial parameters of gait.

This work was part of a larger study that examined differences in kinematic and kinetic gait parameters in children with autism versus a control group [7]. As a result, the combined use of camera-based systems and force plates was required versus other methods of obtaining temporal-spatial measures.

### 2.3. Procedures

Data collection occurred at the motion analysis laboratory at the University of New Brunswick (UNB). A reflective marker was placed directly on the posterior aspect of the right and left calcaneus at the Achilles tendon attachment [19]. Several “warm-up” trials were conducted to allow the participants to adjust to the markers and the laboratory environment. Children were then encouraged to perform as many gait trials as possible. For reliability, at least 7 trials were recorded for each leg. Trials consisted of walking a distance of 25 ft along an unmarked walkway. The physiotherapist positioned each participant at the start line while the parent of the participant waited at the end of the walkway. The start line was located 12.5 ft from the center of the plates allowing each participant to achieve steady state velocity by the time they reached the plate area (recording area). To promote successful force plate strikes with the foot (i.e., one entire foot on a single plate only), the physiotherapist adjusted the starting position of each participant. Verbal instructions to each participant included “look at your mom/dad” (to prevent the child from noticing and potentially targeting the force plate area) and “just walk” (to prevent running). Each trial was approximately 8 seconds in duration (time required to walk 25 ft), and the test session lasted approximately 45–60 minutes. It was anticipated that the experimental procedures could be challenging for the autism group due to skin sensitivities and an unfamiliar laboratory environment. However, the presence of family members and a physiotherapist with extensive experience with children with autism at each data collection session facilitated successful gait measurements and adherence to verbal requests.

### 2.4. Data Analysis

**Figure 1:**
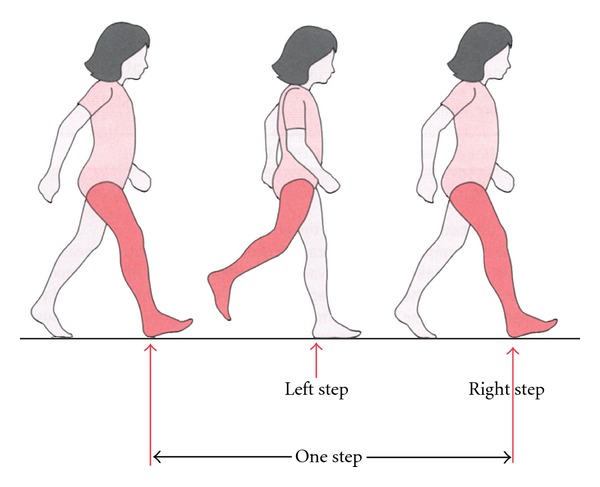
One gait cycle, or stride, is defined as two consecutive heel strikes of the same limb. Each gait cycle is composed of two steps. Reprinted with permission: Elsevier Books (2857691435547).

**Figure 2:**
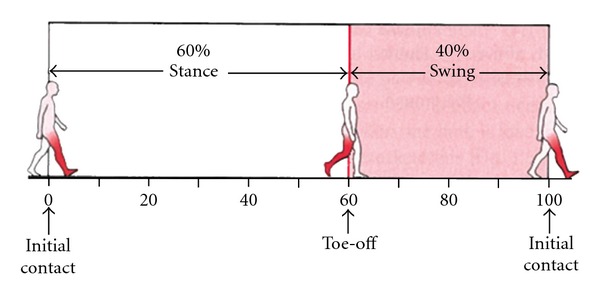
The gait cycle can be divided into the stance phase (weight-bearing) and swing phase (non-weight-bearing). Reprinted with permission: Elsevier Books (2857691435547).

Data was exported from the Vicon software to Matlab (The Mathworks, Natick, MA, R13) for processing. Temporal-spatial variables were computed for each participant's left and right leg gait cycles. A gait cycle is defined as the time between consecutive foot strikes of the same limb. Each gait cycle is comprised of two steps and one stride (Figure 1). The gait cycle can be subdivided into the stance phase and swing phase. The stance phase refers to the weight-bearing portion of the gait cycle for a single limb and typically occurs for the first 60% on average (Figure 2). The swing phase refers to the non-weight-bearing portion of the gait cycle for a single limb and typically occurs for the last 40% of the gait cycle (Figure 2).

**Figure 3:**
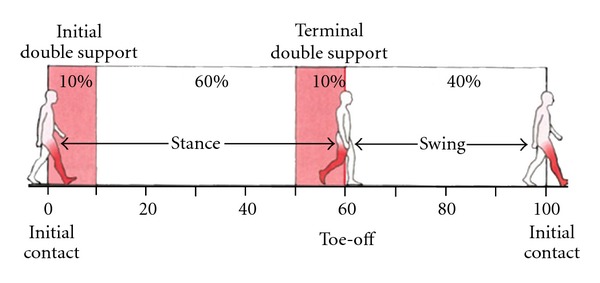
The gait cycle has two periods of double support, where both feet are weight-bearing. Reprinted with permission: Elsevier Books (2857691435547).

For each left and right limb gait cycle, the following temporal-spatial variables were computed:

1. cadence (steps/min): number of steps taken per minute (cadence = 120/cycle time, where cycle time is the duration of the gait cycle),
2. stride length (m): absolute horizontal distance traveled by the heel marker between two consecutive foot strikes of the same limb (Figure 1),
3. swing time (s): duration of time that the limb is not in contact with the floor (Figure 2),
4. stance time (s): duration of time that the limb is in contact with the floor (Figure 2),
5. double stance time (s): duration of time that both feet are in contact with the floor (Figure 3),
6. swing/stance ratio: ratio of swing and stance time.

To identify the most suitable trial for analysis, cadence, velocity, and percent of cycle spent in single stance (i.e., single limb support) were computed for the left and right leg of each participant. The single left and right gait cycle that most closely approximated the respective mean of all gait cycles on these three measures was selected for final analysis for each participant.

For each temporal-spatial measure (TS), six measures of symmetry were computed:

1. symmetry ratio, ratio = TS_right_/TS_left_,
2. symmetry index average, SI_avg_ = [(TS_right_ − TS_left_)/0.5(TS_right_ + TS_left_)] × 100%,
3. symmetry index left, SI_left_ = [(TS_right_ − TS_left_)/TS_left_] × 100%,
4. symmetry index right, SI_right_ = [(TS_right_ − TS_left_)/TS_right_] × 100%,
5. symmetry angle [15], SA = [(45° − atan(TS_right_/TS_left_)) × 100%]/90,
6. gait asymmetry [16], GA = |100 × [ln⁡(TS_right_/TS_left_)]|.

### 2.5. Statistical Analysis

Six symmetry indices were computed using 6 different temporal spatial measures, for a total of 36 symmetry measures. Significant (*P* < 0.05) differences in the six mean temporal-spatial parameters (Table 2) between the control and autism groups were tested using a MANOVA. Significant (*P* < 0.05) differences in the mean symmetry indices (Table 3) between the control and autism groups were also tested using a MANOVA. All statistical tests were performed using SPSS Software (v17.0, IBM, New York, USA).

## 3. Results

No significant differences (*P* > 0.05) in mean symmetry indices data or mean temporal-spatial data were found between the autism and control group. Descriptive data for the six temporal-spatial measures for the autism and control group are provided in Table 2. No significant differences in mean walking velocity were observed between groups (autism: 99.89 cm/s; control: 101.82 cm/s). Descriptive data for the six symmetry indices for the autism and control group are provided in Table 3.

## 4. Discussion

The purpose of this research was to examine differences in gait symmetry between a group of children with autism and an age, height, and weight-matched control group. Using temporal-spatial data, six different symmetry indices were computed and analyzed using MANOVAs. The results of the statistical analysis showed no significant group differences (*P* > 0.05) for the six symmetry indices for cadence, stride length, swing time, stance time, double stance time, and swing/stance ratio. Further, no significant differences (*P* > 0.05) were observed between groups for the mean value of each temporal-spatial measure.

To date, most studies of gait symmetry in autism have focused on infants and toddlers. Several of these studies have reported gait asymmetries [2, 3, 11, 20]. However, little is known about the persistence of these deviations with development. Based on the results of this study, children with autism walk with a consistent interlimb pattern compared to age-matched controls. Due to a small sample size, further work is needed to validate these results.

Previous studies of gait symmetry in children with autism have typically used walking observation scales and video analysis. To our knowledge, no motion capture analyses of gait symmetry exist for this specific population. However, gait symmetry in adults with autism has been examined using similar techniques. Hallett et al. [10] measured gait in 5 adults (aged 25–38 years) with autism versus a control group [10]. No significant differences were found between limbs in the autism group for the kinematic and kinetic gait variables. As a result, the data for each limb was pooled prior to comparison with the control group. Only the ankle range of motion was found to be significantly different between the adults with autism and the control group, with the autism group showing a smaller range of motion.

The results of this study are specific to the age group, task, and gait parameters selected. In addition, the gait parameters used to compute the indices also play an important role in describing gait symmetry. The present study focused on temporal-spatial data; however, other kinematic (e.g., joint angles) and kinetic (e.g., joint moments) gait parameters may provide insight into gait symmetry in children with autism. Previous research [7] suggests that ankle dorsiflexion angles and sagittal hip and ankle moments differ in children with autism versus age-matched controls. Therefore, gait symmetry should also be examined using these parameters.

As symmetry assessments are sometimes dependent on the specific symmetry index used, the present study computed several indices using temporal-spatial data. Similarly, Patterson et al. [21] used four indices to examine gait asymmetry after stroke. Their results also showed that the indices were similar in discriminative ability, but differed in terms of interpretability. Therefore, symmetry indices, such as ratios, may be preferable for their ease of interpretation.

Symmetry measures can be a useful measure of interlimb coordination and help identify gait compensations. A limitation of indices is that the results do not provide an indication of abnormality. That is, the left and right limb could be equivalently abnormal and therefore symmetrical. However, results of tests for mean differences in temporal-spatial data between groups were not significant. Therefore, it would appear that children with autism have normative temporal-spatial data for the left and right legs.

## 5. Conclusions

No previous studies have examined gait symmetry using motion capture in older children with autism. While studies of movement in infants and toddlers have revealed asymmetries, this study found no significant differences in temporal-spatial data or symmetry indices between the autism group and the age-matched controls. Future work should use larger sample sizes to validate the findings in the present study. Additional studies should also focus on different biomechanical inputs to the gait symmetry indices, including kinematics and kinetics. A greater awareness of movement deviations could be beneficial for treatment planning in children with autism, if needed.

## Figures and Tables

**Table 1:** Participant characteristics for the autism group.

Subject	Age (years)	Height (cm)	Weight (kg)	Diagnoses
Male	5.25	118.5	23.1	No known diagnoses
Female	5.50	113.0	26.5	No known diagnoses
Male	5.83	116.0	25.4	Generalized mild hypotonia
				Gross motor delay
Male	8.42	136.5	45.4	No known diagnoses
Male	5.33	113.0	20.2	Generalized mild hypotonia
Male	5.33	124.0	30.6	Generalized mild hypotonia
Male	5.67	113.0	22.7	Gross motor delay
Male	5.75	108.5	21.3	Joint hypermobility
Male	8.25	135.0	51.9	No known diagnoses
Male	5.91	115.5	22.0	Mild central hypotonia
				Gross motor delay
Female	5.67	99.1	14.5	No known diagnoses
Male	9.08	160.2	48.1	No known diagnoses

**Table 2:** Descriptive statistics (mean and standard error) for temporal-spatial data for the autism and age-matched control group.

Group	Cadence (steps/min)	Velocity (cm/s)	Stride length (m)	Swing time (s)	Stance time (s)	Double stance time (s)	Swing/stance ratio
Control	129.11 (2.45)	101.82 (3.39)	0.95 (0.03)	0.38 (0.01)	0.57 (0.01)	0.19 (0.01)	0.68 (0.01)
Autism	134.27 (3.32)	99.89 (3.14)	0.90 (0.03)	0.36 (0.01)	0.56 (0.02)	0.20 (0.01)	0.65 (0.01)

**Table 3:** Descriptive statistics (mean and standard error) for the symmetry indices for the autism and age-matched control group.

		Ratio		SI_avg_		SI_rlr_		SI_rll_		SA		GA	
		Mean	SE	Mean	SE	Mean	SE	Mean	SE	Mean	SE	Mean	SE
Cadence	Control	1.02	0.01	6.84	0.97	6.59	1.00	7.33	0.96	29.0	0.44	6.88	0.98
	Autism	1.09	0.05	10.57	3.74	9.42	3.08	12.29	4.74	30.72	1.37	10.66	3.80
Stride length	Control	0.98	0.03	11.17	1.86	11.59	1.88	11.12	1.96	27.5	0.81	11.23	1.87
	Autism	0.98	0.03	8.72	1.80	9.03	1.98	8.52	1.70	27.58	0.92	8.74	1.81
Swing time	Control	0.99	0.03	11.79	1.60	12.20	1.75	11.64	1.51	27.9	0.89	11.83	1.61
	Autism	0.93	0.04	12.78	3.41	14.47	4.47	11.68	2.78	25.85	1.36	12.88	3.47
Stance time	Control	1.00	0.02	9.19	1.01	9.45	1.01	9.12	1.02	28.4	0.61	9.22	1.01
	Autism	0.97	0.03	7.70	2.76	8.42	3.27	7.19	2.41	27.22	1.04	7.73	2.78
Double stance time	Control	1.01	0.05	18.00	3.19	19.67	3.17	17.70	3.59	27.9	1.47	18.22	3.23
	Autism	1.00	0.05	14.36	3.02	14.90	3.32	14.23	2.96	28.10	1.58	14.43	3.04
Swing/stance	Control	0.99	0.02	8.52	2.39	8.88	2.75	8.4	2.24	28.02	1.10	8.55	2.42
	Autism	0.96	0.02	10.59	2.36	11.26	2.71	10.13	2.13	27.15	1.13	10.63	2.37
